# Co-Expression and Co-Purification Enable Manufacturing of a Six-Monoclonal Antibody Botulinum Antitoxin Cocktail

**DOI:** 10.3390/toxins18050199

**Published:** 2026-04-23

**Authors:** Andrew Davis, Kamaljit Bajwa, Zachary Martinez, Ryan R. Davis, April Green, Fletcher Suber, Shauna Farr-Jones, Milan T. Tomic

**Affiliations:** 1 Resilience Government Services, 2061 Challenger Drive, Alameda, CA 94501, USA; 2 Resilience Government Services, 13200 NW Nano Court, Alachua, FL 32615, USA; 3 Department of Anesthesia, University of California, 1001 Potrero Ave, San Francisco, CA 94110, USA

**Keywords:** oligoclonal antibodies, botulinum antitoxin, co-expression, co-purification, recombinant antibody manufacturing

## Abstract

A highly potent antitoxin for botulinum neurotoxin (BoNT) serotypes A and B has been developed that comprises three monoclonal antibodies (mAbs) targeting BoNT/A and three targeting BoNT/B. These oligoclonal antibody combinations neutralize toxin by simultaneously binding non-overlapping epitopes, thereby promoting rapid toxin clearance. All six mAbs use the same human Fc and framework and have been individually manufactured using the same expression platform and purification process. To minimize the time and labor required to produce the divalent antitoxin, we tested a co-expression and co-purification strategy for the three mAbs per serotype. The mAbs were expressed in CHO-K1 cells, and the media were optimized for co-expression in 10 L bioreactors. Chromatographic co-purification consisted of Protein A capture, followed by strong anion exchange chromatography in flow-through mode and cation-exchange chromatography in bind-elute mode. Co-expression experiments demonstrated that expression of the three anti-BoNT/A antibodies remained within approximately ±30% of the optimal equimolar ratio, whereas the anti-BoNT/B antibodies showed greater variability. Downstream purification steps achieved recoveries greater than 95% per chromatographic step, resulting in overall process yields of approximately 63–75%. This strategy provided sufficient purity of all six mAbs while largely preserving their relative ratios. These results demonstrate the feasibility of producing oligoclonal antitoxin antibodies using co-expression and shared purification strategies. Such approaches may simplify the manufacturing of antibody cocktails while maintaining product quality and biological activity.

## 1. Introduction

Botulism is a frequently fatal neuroparalytic disease caused by intoxication with botulinum neurotoxin (BoNT). Due to the extreme potency of BoNT and the potential for its misuse as a biological weapon, the National Institute of Allergy and Infectious Diseases (NIAID) and the US Department of Defense have funded the discovery and development of next-generation antitoxins to replace equine-derived antitoxin. Neutralizing monoclonal antibodies have emerged as promising countermeasures because they can provide highly specific toxin neutralization with improved safety compared with equine-derived antitoxin products [1,2,3,4,5]. Recombinant oligoclonal monoclonal antibody antitoxins targeting botulinum neurotoxins have been under development for more than two decades and have demonstrated highly potent neutralizing activity in multiple animal models and early clinical studies [1,2,3,4,5,6,7,8,9]. The mechanism of action of the BoNT-neutralizing antibodies requires simultaneous binding of three antibodies to non-overlapping epitopes on the toxin to facilitate rapid clearance [1,2,3,4,5].

These three-antibody drug products have been manufactured by producing each antibody separately in Chinese hamster ovary K1 (CHO-K1) cells, purifying them individually, and subsequently combining them in equimolar ratios. While this approach allows precise control of antibody composition, strict equimolar ratios are not required for effective toxin neutralization. Because toxin clearance depends on the presence of all three antibodies, the cocktail’s functional potency is primarily determined by the antibody at the lowest concentration. The most potent antitoxin on a mass basis would contain an exact equimolar ratio of the three toxin-binding antibodies. Excess of individual antibodies does not impair the mechanism of action, although it would reduce potency when expressed on a per-mass basis.

This study aimed to evaluate the co-expression and co-purification of three antibodies that comprise botulinum antitoxin drug products targeting single serotypes. The existing antitoxin manufacturing process combines individually purified antibodies, allowing precise formulation of the optimal equimolar ratio of each antibody in the cocktail, but requires multiple upstream and downstream manufacturing campaigns and substantial production resources.

The manufacture of oligoclonal antibody therapeutics presents challenges not encountered in conventional monoclonal antibody production, as each antibody component is typically expressed, purified, and formulated separately before combination. While this approach enables precise control over antibody composition, it increases upstream and downstream processing requirements, the analytical burden, and time and labor required. Platform manufacturing strategies for monoclonal antibodies—typically based on Protein A capture followed by ion-exchange polishing—have been widely adopted to improve efficiency, robustness, and scalability across diverse antibody products [10,11]. Extending these platform concepts to antibody mixtures has been proposed as a means to streamline production, with prior studies describing co-expression, co-purification, or alternative manufacturing approaches for recombinant antibody combinations [12,13]. As the number of oligoclonal and multispecific antibody therapeutics entering clinical development continues to increase, there is growing interest in manufacturing strategies that enable single-batch production while maintaining product quality and consistency [14].

Manufacturing a mixture of antibodies in the same bioreactor has several advantages over producing antibodies individually. The motivation for co-expression is not necessarily increased volumetric productivity but rather the simplification of the manufacturing process. Production of antibody cocktails by separate monocultures requires independent upstream campaigns, purification processes, and subsequent blending of the purified antibodies. Co-expression enables production of the antibody mixture in a single upstream culture followed by a shared downstream purification process, thereby reducing manufacturing complexity, batch records, and analytical testing requirements [15]. Reducing manufacturing complexity reduces manufacturing costs. The overall development costs of single-batch manufacturing are estimated to be about one-third less expensive (see Figure 4 of reference [12]). Co-expression may also reduce variability associated with post-production blending of separately manufactured antibodies by generating the antibody mixture within a single manufacturing batch.

Supply chain issues that were acute during the COVID-19 pandemic [16] provided the impetus for this work, as we sought to use bioreactors and other consumables more efficiently. These considerations motivated the investigation of co-expression strategies that could enable more efficient manufacturing of oligoclonal antibody therapeutics.

Co-expression was evaluated by comparison of three media for expression of the two three-antibody products (i.e., BoNT/A antitoxin, NTM-1631, and BoNT/B antitoxin, NTM-1632). Antibodies used in the clinical studies all have the same constant region framework and were individually expressed in a CHO-K1 cell line, purified, and then combined in equimolar ratios. Three antibody mixtures targeting serotypes A and B of BoNT have completed Phase I clinical trials to prevent BoNT/A botulism (NTM-1631) [6] and BoNT/B botulism (NTM-1632) [7].

Co-purification evaluated the testing chromatography conditions for the six-mAb product (BoNT/A and BoNT/B antitoxin, G03-52-01). G03-52-01 is a mixture of NTM-1631 and NTM-1632 [8,9]. These studies provide proof of principle for labor- and time-efficient production of oligoclonal recombinant botulinum antitoxin. The goal of the studies described here was to determine whether a common expression and purification strategy could be applied to mixtures of antibodies with similar physicochemical properties.

Oligoclonal antibody products across multiple therapeutic areas have been approved by the FDA. These include drugs for infectious diseases [17], antivirals for COVID-19 [18] (casirivimab, imdevimab), and HIV [19], antibody combinations for cancer [20] (e.g., trastuzumab/pertuzumab [21], and ipilimumab/nivolumab [22]); REGN-EB3 oligoclonal for Ebola [23,24]. In the field of botulinum antitoxins, the European-developed AntiBotABE program has also advanced recombinant oligoclonal antibody mixtures targeting botulinum neurotoxin serotypes A, B, and E [25]. Adams et al. have co-expressed four antibodies targeting snake venoms in a co-culture of four CHO cell lines, and the expected antibody titers were obtained [26]. Given its neglected tropical disease status, reducing the production costs of snake antivenom was the primary impetus for the co-expression studies. As the number of oligoclonal antibody therapeutics entering clinical development continues to increase, manufacturing strategies that enable efficient co-expression and purification will become increasingly important. Approaches that enable co-expression and shared downstream purification of multiple antibodies could substantially simplify manufacturing workflows while maintaining product quality and potency.

## 2. Results

The experimental strategy followed three sequential objectives. First, we evaluated whether the antibodies could be co-expressed under common culture conditions and maintain approximately equimolar ratios. Second, we confirmed that the individual antibodies could be analytically distinguished, enabling monitoring of antibody ratios during development. Finally, we investigated chromatographic purification strategies that would allow co-purification of the antibody mixtures while preserving their relative ratios.

### 2.1. Media Selection in Ambr Bioreactors

Prior to testing in large bioreactors, we assessed the feasibility of co-cultivation and selected an optimal culture medium to enhance growth and productivity. Using the Ambr^®^ 15 Cell Culture system (Sartorius, Göttingen, Germany), a 48-bioreactor setup with 10–15 mL capacity each, we controlled variables dissolved oxygen, mixing, temperature, and pH. The study employed a design of experiments (DOE) to test the proprietary XJEM medium, Gibco™ Dynamis™ AGT, and EX-CELL^®^ Advanced Media during the culture of individual anti-BoNT/A and anti-BoNT/B mAb clones and their equimolar combinations.

**Figure 1:**
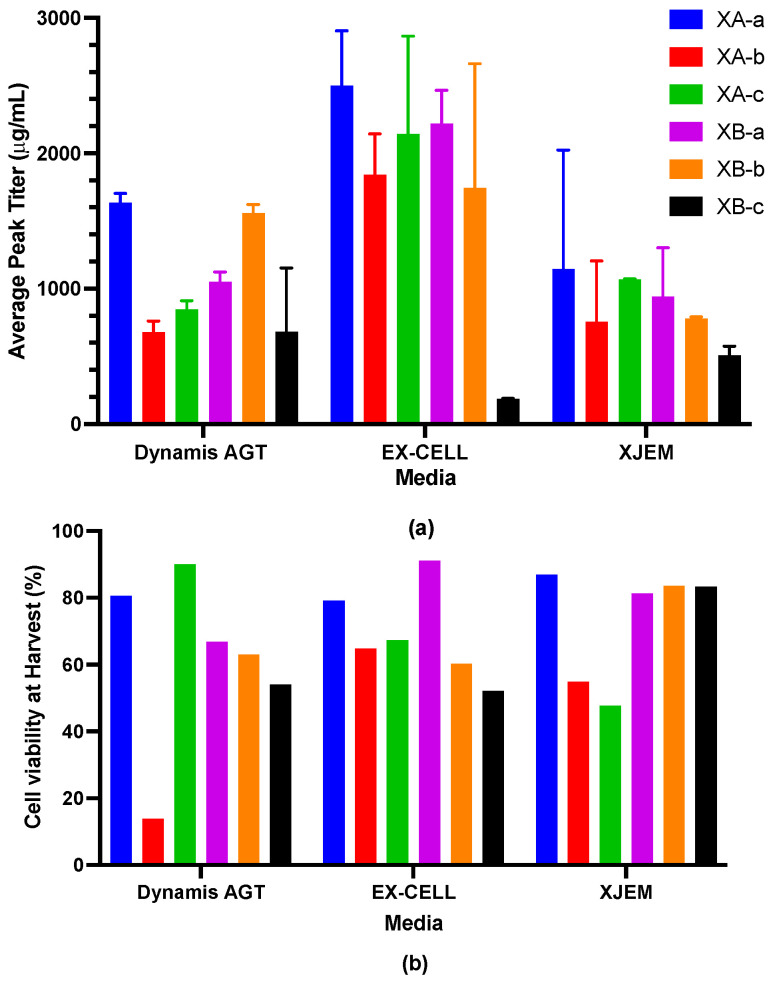
Antibody titer and cell viability in three different media in Ambr cultures of single antibodies. (**a**) Average peak antibody titer (error bars indicate ± SEM) and (**b**) cell viability at harvest. Media tested were Gibco Dynamis AGT, EX-Cell, and XJEM (a proprietary media). Cell viability was measured using trypan blue exclusion. Cultures were performed in duplicate or triplicate.

Cell growth was evaluated by peak viable cell density, and productivity was measured by antibody titer at harvest. Cell viability and the antibody titer were monitored over two weeks. Media was then harvested and frozen prior to analysis. As shown in Figure 1, the commercially available EX-CELL Advanced media provided the highest antibody titers for five mAbs, while XB-c had the lowest titer in all media. However, differences between media as measured by cell viability or antibody titer were not statistically significant. EX-CELL and the proprietary XJEM media had over 50% cell viability at harvest for all six cell lines.

Based on the results of using different culture media for individual cell lines, we then evaluated co-expression of three mAbs in the EX-CELL media and XB-c in the Gibco Dynamis AGT media. The six co-expressed samples and the individually cultured antibody samples were filtered to 0.2 µm and purified on Protein A using the ÄKTA Explorer 100 (Cytiva, Uppsala, Sweden). EX-CELL Advanced media (Sigma Aldrich, St. Louis, MO, USA) was used for the co-expression experiments because it produced the highest titers in individual mAb cultures.

The Ambr run supernatants were loaded on Protein A column to purify the antibodies. As shown in Table S1, the antibody recovery was reasonable with negligible antibody loss in the flowthrough and wash pools. Elution fractions were pooled based on A280 nm measurement on a Nanodrop (Thermo Fisher, Waltham, MA, USA). The BoNT/A mAb recoveries of the co-expression antibodies are 10 to 20% higher than the BoNT/B mAb co-expression antibody recoveries.

**Figure 2:**
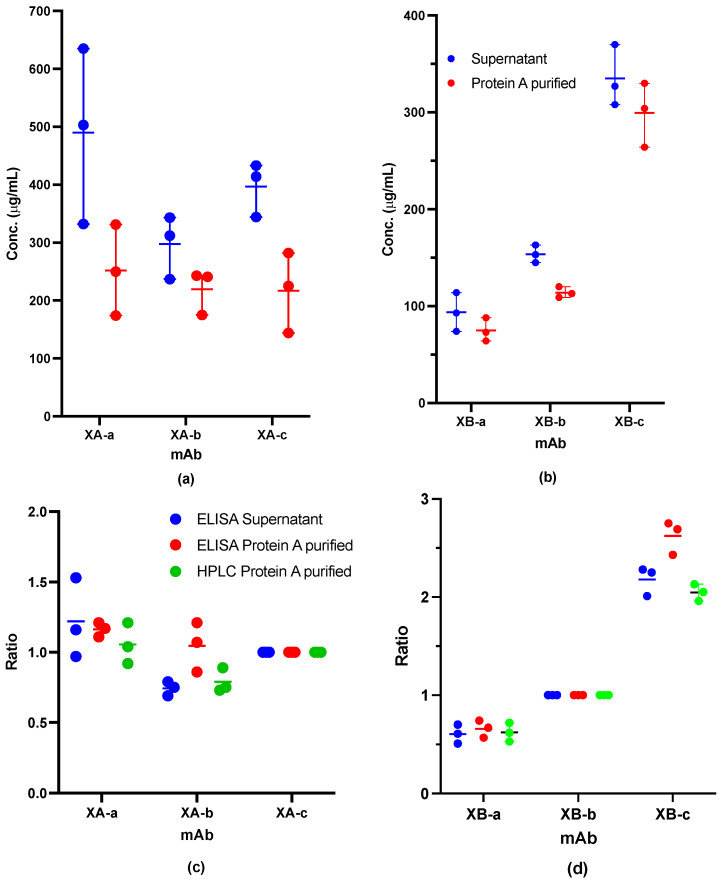
Concentrations and relative antibody ratios from Ambr co-expressions were determined using ELISA. (**a**,**b**) Concentrations from three measurements of cell culture supernatant following centrifugation and following Protein A purification as determined by ELISA for (**a**) anti-BoNT A mAbs and (**b**) anti-BoNT/B mAbs. (**c**,**d**) Relative concentration ratios of each component mAb from either cell culture supernatant by ELISA (shown in blue); from Protein A purification by ELISA (shown in red) or by HPLC (shown in green). For anti-BoNT/A mAbs, the concentration ratio was relative to XA-c; (**c**) for anti-BoNT/B mAbs, the concentration ratio was relative to XB-b (**d**). Each dot represents an individual measurement. The horizontal line indicates the average of the three measurements.

ELISA was performed on the clarified supernatants from the Ambr co-expressions and the material purified on Protein A, following IEX-HPLC separation. ELISA and the IEX-HPLC quantitation of the mAbs are shown in Figure 2c,d. Overall, we observed expression of all the XA mAbs in the co-expression to be within 30% of the optimal 1:1:1 ratio. The XB mAbs showed a wider variation, varying from 50% to 250% when compared to the normalized mAb. As expected for mixed-culture systems, individual antibody titers in co-expression cultures were somewhat lower than those observed in monoculture. However, all antibodies remained detectable and within ranges suitable for downstream purification and formulation. Importantly, the relative antibody ratios measured in the clarified supernatant and after Protein A purification were similar, indicating that the capture step did not substantially alter the antibody composition.

### 2.2. Three Antibody Co-Purification

**Figure 3:**
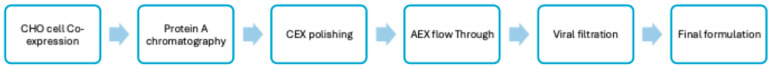
Manufacturing workflow for co-expression and co-purification of oligoclonal BoNT antitoxin antibodies.

A platform chromatographic purification method to co-purify three antibodies was developed based on the method used to purify antibodies individually for Drug Products for NTM-1631 [6], NTM-1632 [7], NTM-1633 [27], and NTM-1634 [28]. The platform purification method we developed is summarized in Figure 3.

To evaluate the above co-purification process, an analytical method that could resolve all antibodies was required. We used a previously established weak cation-exchange (WCX) chromatographic analytical method, developed to characterize nine-mAb co-formulation of anti-BoNT/A, /B, /E [29]. This WCX method establishes a rigorous determination of stoichiometric ratios and biological control that the FDA requires.

Peaks for each antibody on the WCX Chromatograms for the combined BoNT A mAbs and BoNT B mAbs were well resolved, yet impurities were seen, indicating that additional orthogonal chromatographic steps were required. Peaks were assigned by WCX chromatography of individual antibodies run separately (Figure S1).

To evaluate chromatographic behavior of antibodies during process development, mixtures of individually purified antibodies were initially used as standards. This approach ensured that the antibody mixture composition was known and enabled evaluation of chromatographic performance independent of upstream expression variability. Having an assay to measure the amounts of each mAb in the co-formulated mixture, we then were able to optimize the purification scheme, described below.

### 2.3. Comparison of Protein A Capture Materials

We first compared traditional resin and nanocellulose for the Protein A purification step. Two Protein A resin types were evaluated: Cytiva on agarose bead, and the Cytiva Fibro PrismA nanocellulose media in which Protein A is conjugated to electrospun nanocellulose fibers that facilitate convective flow instead of diffusive flow. Previously, we reported that mAb capture productivity on batch and continuous downstream processing was improved using Fibro PrismA nanocellulose compared to agarose beads [30] with a single antibody. The BoNT mAbs used for the comparison of Protein A purification materials were supernatants from cultures produced in 5 L shake flasks. The supernatant was filtered through a 0.22 µm filter before loading onto a 5 mL MabSelect PrismA column (Cytiva, Uppsala, Sweden) with a residence time of 2 min, or a 0.4 mL Fibro unit with a residence time of 2.4 s. Fibro Prism A showed higher productivity (Table S2), and superior clearance of host cell proteins and nucleic acids (Table S3) [30].

### 2.4. Comparison of Cation Exchange Resins for Individual Cultures

Following Protein A capture, the Fibro eluate and PrismA eluate was pooled, held at pH 3.5 for 1 h to inactivate viruses. This eluate was then used to compare cation exchanges resins. First, the pH of the eluate was then raised to pH 5.0 in preparation for loading onto the cation exchange (CEX) columns. Two CEX resins were evaluated. The first was a weak cation exchanger (MilliporeSigma Eshmuno CP-FT resin, Sigma Aldrich, Darmstadt, Germany) and the second was a strong cation exchanger (Cytiva Capto S resin, (Cytiva, Uppsala, Sweden)). The loading capacities were evaluated up to 1000 g/L with a two-minute residence time using a 1 mL column.

The CEX columns had loading capacities ranging from 500 g/L to 1000 g/L with no apparent breakthrough in aggregates. The higher loading capacities resulted in higher recoveries. Recovery and productivity are shown in Table S4, and CEX chromatograms are provided in Figure S2. Eshmuno CP-FT showed slightly higher recoveries (86 ± 6%) compared with Capto S (81 ± 4%).

### 2.5. Comparison of Anion Exchange Resins

The CEX flow-through fractions were then pooled and the pooled fractions were then used to optimize the anion exchange step. Pooled fractions were divided into two aliquots in preparation for the conditioning requirements for the anion exchange (AEX) or mixed mode chromatography (MMC). A 1 mL Cytiva Capto Q column was used for AEX, and a 1 mL Cytiva Capto Adhere column for MMC. The Capto Q load conductivity was set at a maximum of 10 mS/cm. The Capto Adhere load conductivity was targeted at 30 mS/cm. A broad loading capacity was evaluated on the MMC and AEX steps, ranging from 270 g/L to 700 g/L. Productivity and nucleic acid clearance were similar for the two resins, whereas recovery was significantly higher with the Capto Q resin for each mAb (Table S5).

### 2.6. Individual mAb Cultures in 10 L Bioreactors

After identifying a purification process with sufficiently high yield and purity, we then used common expression conditions for each mAb separately at the 10 L scale prior to co-expression. These 10 L runs of single mAbs were used to assess scalability and maintenance of culture conditions, which were prerequisites for co-expression. Each cell line was separately grown in 10 L in bioreactors, initiated with a vial thaw of each of the six clones into EX-CELL^®^ Advanced^™^ CHO Fed-Batch Media (EX-CELL^®^ media, Sigma Aldrich, Darmstadt, Germany). The cell vials used for each of these 10 L runs were from research cell banks (RCB). Clones underwent three to five passages before inoculating the bioreactor. The process parameters, including previously established criteria, are listed in Table S2. The sterile hold for each 10 L run using EX-CELL^®^ media was initiated prior to inoculation at 36.5 °C for 12 h or longer. Glucose was fed as needed, based on sample measurement, and feedings were every 48 h beginning on Day 3 post-inoculation.

Cell suspension sample taken from the reactor was transferred to a 1.5 mL microcentrifuge tube and centrifuged at 1000× *g* for 2 min. The supernatant was transferred to the Octet 96-well plates for analysis.

A temperature shift from 36.5 °C to 32 °C was performed on Day 7 post-inoculation. Three different feeding strategies were implemented in each run: EX-CELL Advanced CHO Feed 1, glutamine, and glucose.

The cell growth and viability trends were consistent with typical CHO cell growth patterns in a fed-batch process. The cells entered an initial lag phase after introduction to the bioreactor. They grew exponentially, doubling until the maximum cell density was reached. Cell viability varied slightly between runs (88.8–69.1%), (Table 1) with XA-c showing the lowest cell viability prior to harvest (69.1%).

Lactate concentration, ammonium ion concentration, pH, and dissolved oxygen were typical of CHO cell cultures and were comparable for all six cell lines. Lactate, a metabolic by-product during exponential growth, increased during the early growth phase in each bioreactor run and subsequently declined as cells shifted toward lactate consumption. In cultures of XA-a, XA-b, and XA-c, lactate concentrations increased to approximately 5.7–6.1 g/L before the metabolic shift to lactate consumption occurred. In contrast, cultures of XB-a and XB-b reached lower peak lactate levels of approximately 2.7–3.1 g/L before entering the consumption phase.

Ammonium ions produced during glutamine consumption provide an additional indicator of culture conditions. As shown in Table 1, the peak ammonium ion concentration was below 10 mM in all cultures except XB-a, which reached 15.6 mM. At harvest, ammonium concentrations ranged from 2.49 to 11.03 mM across the six cultures.

These metabolite profiles indicate that the six CHO cell lines exhibited broadly similar metabolic behavior under the selected culture conditions.

### 2.7. Purification at 10 L Scale for Individual Clones

The 10L bioreactors containing individual clones were harvested using depth filtration media. The diafiltration media were 3M 05SP01A and 60ZB05A grades in a 1:1 surface area ratio at 100 L/m^2^ loading capacity. Pressure readings during operation remained low (<30.3 kPa), indicating that the clarification capacity limit of this depth filter train had not been reached.

Protein A capture step was performed using a 1 L MabSelect SuRe column (Cytiva, Uppsala, Sweden). Due to supply chain constraints that limited the availability of PrismA resin and Fibro units, MabSelect SuRe was used instead, although PrismA was found to provide higher yields. The column was operated with a residence time of 4 min and a maximum loading capacity of 35 g/L. Following Protein A elution, the eluate had an average pH of 4.0 and was adjusted to 3.5 during the viral inactivation step.

Based on results from the 5 L shake flask studies, a 5 mL Eshmuno CP-FT column was selected for the cation-exchange (CEX) step. The column was operated with a loading capacity of 750 g/L and a residence time of 2 min. Recovery exceeded 93% for all clones except XB-b, which was recovered at 88%. Performing the CEX step prior to anion-exchange chromatography (AEX) enabled direct transfer of the material between steps without adjusting conductivity. Recoveries following the AEX step were approximately 100%, within measurement error, for all clones.

Viral reduction was performed using a Viresolve Pro Modus 1.1 viral reduction filter (VRF, Sigma Aldrich, Darmstadt, Germany), with recoveries of approximately 100% for all six clones. Flux decay was ≤30% in five cultures, consistent with expected loading capacities. In contrast, XA-a showed a higher flux decay of 85%, resulting in a reduced loading capacity on the VRF.

Final formulation was performed using a 0.1 m^2^ tangential flow filtration (TFF) cassette, which was slightly oversized for the available material from each clone. Recovery from this step ranged from 88 to 94%. The final 0.22 µm sterilizing filtration step was also oversized and resulted in a measurable decrease in yield following filtration of the drug substance.

Overall process yields were 63–75%, with individual unit operations typically achieving recoveries of ≥95%. The resulting drug substance met specifications for host cell protein and residual Protein A for all clones.

### 2.8. Six Antibody Co-Purification

The co-purification process for the six BoNT/A and /B mAbs that comprise G03-52-01 (i.e., NTM-1631 and NTM-1632) started with a mixture of previously purified antibodies. We used anion- and cation-exchange chromatography (AEX and CEX), which separates based on antibodies’ isoelectric points (pI). Theoretical pI values for the antibodies were XA-a, 7.9; XA-b, 8.2; XA-c 8.4; XB-a, 8.4; XB-b, 8.3; XB-c 8.5. Criteria for the success of co-purification were high recovery and maintenance of the antibody ratios.

To evaluate the proposed strategy, approximately 5 mg of each purified BoNT/A and BoNT/B antibody was dialyzed using a 10 kDa molecular-weight cutoff cassette and buffer-exchanged into AEX equilibration buffer (25 mM citrate, 50 mM NaCl, pH 6.0). The antibodies were then combined in approximately equimass ratios (~3 mg of each antibody). Analytical HPLC chromatograms of the individual antibodies are shown in Figure S3, demonstrating that the six antibodies could be resolved and identified under analytical ion-exchange conditions.

The six-antibody mixture was then loaded onto a strong AEX (Q FF) chromatography column, and the chromatograms of the six mAbs, compared with the cGMP-manufactured G03-52-01 Drug Product reference, were superimposable (Figure S4).

**Figure 4:**
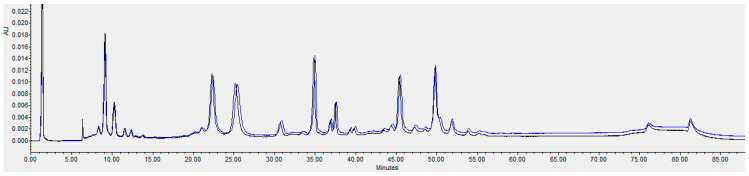
Q flow-through mode (AEX) of the six mAbs. The loaded sample is shown in black and the flow through is shown in blue.

HPLC analysis of the single flowthrough peak collected from the Q column is shown in Figure 4. Recovery of the antibodies was high as seen by comparing the peaks in the flowthrough and loaded sample.

**Figure 5:**
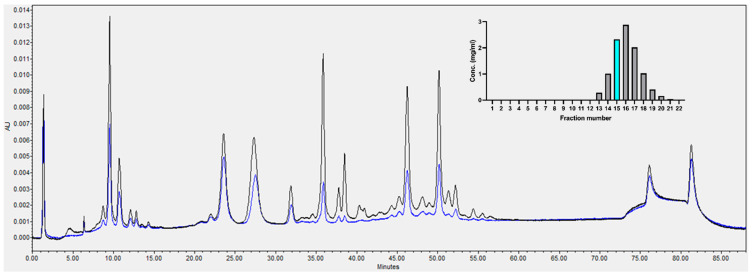
IEX HPLC Analysis of 5 mL HiTrap Capto S Column Fraction 15 (blue) and S column load (black). The inset shows the protein concentration measured by A280 on the Nanodrop spectrophotometer for the collected peak fractions (1 mL per fraction). Fraction 15 in the histogram is highlighted in blue. (IEX-HPLC of individual purified antibodies is shown in Figure S2.) The column was loaded with 20 μg antibody.

After Q flow-through chromatography, 12 mg of the six mAb mixture in 2.5 mM citrate, 5 mM NaCl pH 5.16 (1.35 mS/cm) was loaded on a 5 mL HiTrap Capto S column (Cytiva, Uppsala, Sweden) and eluted with 0–50% of buffer B (10 mM citrate, 2 M NaCl, pH 5.5). A single elution peak was observed. Figure 5 shows the protein concentration from A280 nm measured by the Nanodrop spectrophotometer (Thermo Fisher, Waltham, MA, USA) for the collected peak fractions (1 mL per fraction) and the IEX chromatogram of fraction 15. Note that in the IEX chromatogram, peak heights are influenced by the specific ion-exchange behavior of each antibody and therefore do not directly reflect expression levels or molar ratios.

At pH 5.2–5.5, all six antibodies are positively charged and eluted from the S column as a group in a single peak. Therefore, by applying appropriately selected operation conditions, co-purification of multiple antibodies with high recovery can be achieved, and the relative antibody ratios in the purified product could be maintained.

**Figure 6:**
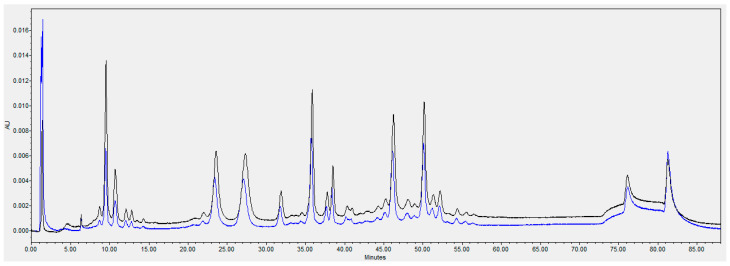
IEX HPLC comparison of CM column load and elution fraction 18. Column load is shown in black, and fraction 18 is shown in blue. The column was loaded with approximately 20 μg of antibody. Detection was at 280 nm. (IEX-HPLC of individual purified antibodies is shown in Figure S2.).

After Q flow-through chromatography, 12 mg of the six-mAb mixture in 2.5 mM citrate, 5 mM NaCl, pH 5.16 (1.35 mS/cm) was loaded on a 5 mL HiTrap CM FF column and eluted with 0–50% of buffer B (10 mM citrate, 2 M NaCl, pH 5.5). Fraction analysis confirmed that the peak fractions contained most of the recovered antibodies (Table S6). The IEX HPLC chromatogram of fraction 18 is shown in Figure 6. Co-purification of the six mAbs was also achieved with high recovery on both the CM column and the Capto S-column.

The IEX-HPLC results demonstrate that ion-exchange chromatography conditions can be selected that enable efficient co-purification of the six BoNT/A and BoNT/B antibodies while maintaining high recovery and preserving relative antibody composition.

## 3. Discussion

Previous oligoclonal antibody therapeutics for BoNT have been manufactured by expressing each monoclonal antibody independently, followed by purification and formulation into a defined mixture. This approach has been used for monovalent botulinum antitoxin cocktails (for NTM-1631 [6], NTM-1632 [7], NTM-1633 [27], and NTM-1634 [28], G03-52-01 [8]) as well as for other oligoclonal antibody therapeutics, including antibody combinations targeting viral infections or cancer. While this strategy enables precise control of antibody ratios, it requires multiple upstream cell culture runs and independent purification campaigns for each antibody, thereby increasing manufacturing complexity, and labor. The emergence of oligoclonal and multispecific antibody therapeutics has highlighted the need for efficient manufacturing strategies, and prior studies have demonstrated that appropriately engineered antibody mixtures can be co-expressed and produced using shared upstream and downstream processes while maintaining developability and scalability [13].

The results of the present study demonstrate that co-expression of multiple antibodies in a single bioreactor, followed by shared downstream purification, can provide a viable alternative manufacturing strategy for oligoclonal antibody products when the antibodies share the same or similar Fc regions and similar physicochemical properties. Antibody mixtures with substantially different physicochemical properties, such as isoelectric point may require additional process optimization.

The three anti-BoNT/A antibodies in our system maintained ratios within approximately ±30% of the most efficient equimolar composition, and downstream purification steps achieved recoveries greater than 95% per chromatographic step with overall process yields of approximately 63–75%. These results compare favorably with conventional monoclonal antibody manufacturing processes while substantially reducing the number of required bioreactors, purification operations, and batch records [26,31]. The primary advantage of this strategy lies in reducing manufacturing complexity rather than maximizing volumetric productivity.

In addition, the purification strategy described here employs a platform sequence of Protein A capture followed by cation-exchange polishing and anion-exchange flow-through chromatography. Because the six antibodies share similar Fc frameworks and isoelectric points, they can be purified together without significant alteration of their relative ratios. This simplifies downstream processing and demonstrates that co-purification strategies may be broadly applicable to oligoclonal antibody products in which component antibodies have similar structural and physicochemical properties.

Small-scale studies using the Ambr cell culture system demonstrated the feasibility of co-expression of three monoclonal antibodies targeting either BoNT/A or BoNT/B. These experiments showed that three antibody-producing CHO cell lines could be cultured together while maintaining robust cell growth and antibody production. Subsequent 10 L bioreactor studies further demonstrated that the same culture conditions could be applied to all six anti-BoNT/A and anti-BoNT/B monoclonal antibodies to achieve reproducible expression levels.

As expected for mixed-culture systems, individual antibody titers in co-expression cultures were somewhat lower than those observed in monoculture. However, all antibodies remained within ranges suitable for downstream purification and formulation.

Although the co-expression experiments showed variability in the relative ratios of the three antibodies, precise equimolar ratios are not required for antitoxin activity. Neutralization of BoNT requires simultaneous binding of three antibodies to distinct epitopes on the toxin, and therefore, overall potency is primarily determined by the antibody present at the lowest concentration. Variability in the ratios of individual antibodies is therefore expected to have a limited impact on the functional activity of the antibody cocktail, provided that all three antibodies are present at sufficient levels. In natural polyclonal immune responses, antibodies targeting different epitopes of the same antigen are not present in equimolar ratios. Therapeutic antibody cocktails are typically formulated at equimolar ratios primarily to maximize neutralizing potency on a per-mass basis and to simplify dosing, rather than because strict stoichiometric equivalence is required for biological activity.

Downstream purification studies demonstrated that the same purification conditions could be applied to all six antibodies. The purification strategy consisted of Protein A capture followed by cation-exchange chromatography (Eshmuno CP-FT) and strong anion-exchange chromatography (Capto Q). These steps were selected based on their ability to remove impurities while maintaining high recovery across antibodies with similar physicochemical properties.

Analysis of the chromatographic fractions showed that all six antibodies co-purified efficiently in both the AEX and CEX steps. In the AEX flow-through mode and the CEX bind-and-elute mode, the antibodies eluted as single dominant peaks, indicating effective co-purification. Although fraction analysis revealed modest differences in elution profiles among individual antibodies—reflected in changes in peak area in early and late fractions—the overall chromatographic peaks remained well defined and undistorted. Importantly, the relative antibody ratios in the purified product were largely preserved compared with the input mixture, indicating that the ion-exchange processes did not significantly bias the antibody cocktail’s composition.

Downstream processing of the six antibodies produced in 10 L bioreactors demonstrated successful purification using a three-step chromatographic process with average recoveries exceeding 95% per step. The efficiency of this process resulted in an overall process yield greater than 70%, indicating that high recovery can be maintained while applying a common purification scheme to multiple antibodies. The resulting drug substance met all analytical specifications using identical process parameters and loading capacities for each monoclonal antibody clone.

These results demonstrate that co-expression and shared purification of multiple monoclonal antibodies is feasible when the antibodies share similar Fc regions and physicochemical properties. Although individual antibody titers in co-expression cultures may be lower than those obtained in monocultures, the manufacturing advantages arise from simplifying the overall process rather than from increased volumetric productivity. Such approaches may simplify the manufacturing of oligoclonal antibody therapeutics by reducing the number of upstream cultures, purification campaigns, and batch records required compared with conventional strategies in which each antibody is produced and purified separately. Simplification of manufacturing, purification, batch records and analysis requires significantly less labor than culturing mAbs separately (see analysis of time and labor requirements in Table S9 and Figure S5).

Future process characterization studies will evaluate selected process parameters to further optimize the platform. Areas for improvement include reducing residual host cell proteins in XA-a and XA-c and increasing the proportion of monomeric species in XA-c. Additional work will also be required to evaluate process robustness, scalability, and long-term control of antibody ratios during larger-scale manufacturing.

## 4. Conclusions

The primary objective of this work is to demonstrate feasibility, specifically answering the questions: (1) can multiple antibodies be co-expressed under the same conditions, (2) can co-expressed antibodies be fully resolved and quantified using commonly deployed analytical techniques, and (3) can co-expressed antibodies be purified while maintaining stoichiometric ratios and biophysical characteristics?

Results presented here demonstrate the feasibility of co-expression of three monoclonal antibodies and co-purification of six-antibody mixtures targeting botulinum neurotoxin serotypes A and B. Implementing co-expression and shared downstream purification reduces manufacturing complexity by decreasing the number of required upstream cultures, purification campaigns, and associated batch records, thereby improving production efficiency and potentially reducing manufacturing costs resulting from additional labor. Such approaches may provide practical advantages for the manufacturing of antibody combinations. Future work would include a direct, side-by-side comparison of co-expression vs. individual expression to determine scalability, and robustness of cell line expression over time and to quantify the economic benefits of co-expression.

The findings reported here may be broadly generalizable to antibody products comprising multiple antibodies with shared Fc frameworks and similar physicochemical properties and have the potential to reduce the facility and labor requirements for antibody combination drug products.

## 5. Materials and Methods

### 5.1. Materials

#### 5.1.1. Cell Lines

Cells for production were from working cell banks of CHO-K1 cell lines used to produce drug substances comprising NTM-1631 [6] and NTM-1632 [32].

#### 5.1.2. Media

Three different cell culture media were used: two commercially available EX-CELL^®^ Advanced medium for CHO Cells (Sigma-Aldrich, St. Louis, MO, USA), Gibco ^TM^ Dynamis ^TM^ AGT ^TM^ Medium (Thermo Fisher, Waltham, MA, USA), and XJEM, a proprietary cell culture medium.

#### 5.1.3. Antibodies

Six IgG1 antibodies made individually under cGMP and formulated as a liquid at 5 mg/mL concentration were used as reference materials. These six antibodies were: XA-a, XA-b, XA-c, the components of NTM-1631 [6]; and XB-a, XB-b, XB-c, the components of NTM-1632 [32].

#### 5.1.4. Chromatography and Purification Materials

Cytiva HiTrap Capto MMC, Cytiva MabSelect PrismA, Cytiva Fibro PrismA, Eshmuno CP-FT, Cytiva Capto Q (1 mL), Cytiva Capto S (1 mL), Cytiva Capto Adhere (1 mL), MabSelect SuRe resin, MilliporeSigma ViResolve Modus 1.1 viral reduction filter (Sigma, St. Louis, MO, USA), Pall (Port Washington, NY, USA) Cadence Omega 0.1 m^2^ 30 kDa TFF cassette.

### 5.2. Cell Culture in Ambr Bioreactors

The Ambr^®^ 15 automated microbioreactor system (Sartorius, Göttingen, Germany) has 48 parallel cultivation bioreactors with a 10–15 mL scale. Midpoint process parameters for the first DOE study were: Seed density (Vc/mL) 0.4 × 10^6^, pH 7, dissolved oxygen (DO) 20%, rotations per minute 500, temperature 36.5 °C shifted down to 32 °C. Parameters for the DOE power calculations are shown in Table 2.

Two or three replicates were tested as shown in Table 3.

Purification was performed in two steps: Protein A affinity chromatography followed by ion exchange chromatography.

The EX-Cell Adv CHO Ambr run supernatants were loaded on Protein A column (Cytiva 17549852, HiTrap™ MabSelect™ PrismA, 1 mL) to purify the antibodies (equilibration/wash buffer: 20 mM sodium phosphate, 150 mM NaCl, pH 7.20; elution buffer: 0.1 M citrate, pH 3.50; neutralization buffer: 1 M Tris, pH 9.0). Elution fractions that contained antibody were then pooled based on A280 nm measurement on Nanodrop spectrophotometer (ThermoFisher, Waltham, MA, USA).

### 5.3. Cell Culture in 5 L Shake Flasks

BoNT mAbs used for the comparison of MabSelect PrismA and Cytiva Fibro PrismA and were produced in 5 L shake flasks with 2 L of EX-CELL media per flask. Samples were taken before adding 150 mL of concentrated feed. The final target concentration of glutamine was 4 mM, and the final target concentration of glucose was 8 g/L. Cells were harvested on Day 11 or 12. The shake flasks were harvested by centrifugation at 5000× *g* for 30 min at room temperature. The supernatant was filtered through a 0.22 µm filter before loading onto the two different Protein A units.

### 5.4. Cell Culture in 10 L Bioreactors

BioBLU^®^ 10c bioreactors (Eppendorf US, Enfield, CT, USA) were used to determine optimal growth and feeding strategies. The upstream process for each 10 L run was initiated by thawing a vial of each clone into EX-CELL^®^ Advanced^™^ CHO Fed-Batch Media (EX-CELL^®^ media). Research cell bank (RCB) vials were used for each 10 L run. Clones underwent three to five passages before being inoculated into the reactor. The bioreactor process parameters are shown in Table S6. The sterile hold for each 10 L run using EX-CELL^®^ media was initiated prior to inoculation at 36.5 °C for ≥12 h. Glucose was fed as needed, based on sample measurement, and EX-CELL Advanced CHO Feed 1 feedings were scheduled every 48 h beginning on Day 3 post-inoculation. Metabolite readings, cell counts, and Octet analyses were performed daily. A temperature shift (from 37 °C to 32 °C) occurred for each run, seven days post-inoculation. XA-a, XB-a, and XB-c were harvested 14 days post-inoculation, XA-b and XB-b were harvested 13 days post-inoculation, and XA-c was harvested 11 days post-inoculation.

Metabolite readings, cell counts, and mAb titer were performed daily beginning on Day 3 post-inoculation. For sampling, at least 20 mL of culture was removed with a syringe to deprime the sample line, and a 5 mL sample was then taken for the analyses. Metabolites, pH, and partial pressures of O_2_ (PaO_2_) and CO_2_ (PaCO_2_) were measured using Bioprofile^®^ FLEX2 (Nova Biomedical, Waltham, MA, USA), and antibody titer was measured on Octet^®^ BLI Detection System (Sartorius).

### 5.5. Cell Viability Measurements

Cell viability as measured using trypan blue exclusion on a Vi-CELL™ Cell Viability Analyzer (Nexelcom Bioscience LLC, Lawrence, MA, USA). Viable cell density was measured on Vi-CELL BLU cell analyzer (Beckman Coulter, Brea, CA, USA).

### 5.6. Analytical Ion Exchange (IEX) Chromatography

Pooled fractions from Protein A chromatography were injected onto a guard column Opti-SOLV mini filter (Sigma Aldrich, St. Louis, MO, USA), 0.5 μm (Injection volume 20 μL) at a flow rate of 0.8 mL/min. The analytical column was Pro-PAC^TM^ WCX-10 (Thermo Scientific, Waltham, MA, USA) 4 mm × 250 mm, 10 μm. The mobile phase gradient was as listed in Table S7 with mobile phase A 10 mM sodium phosphate pH 6, and mobile phase B 0.3 M sodium phosphate, pH 7.3.

### 5.7. ELISA for Quantification of Individual Antibodies in the Mixtures

The ELISA detects individual antibodies, alone or in a mixture with other antibodies, using BoNT domains specific to each antibody as described in [33] for serotype A antibodies and in [32] for serotype B antibodies. Briefly, anti-BoNT antibodies were bound to microtiter plates, followed by antibody-specific domains. Indirect capture was used to avoid avidity effects. The mixture of mAbs to be tested was then added to the wells, and binding was detected using a mouse anti-SV5 antibody and HRP-coupled anti-mouse mAb.

## Figures and Tables

**Table 1:** Cell viability and ammonium concentrations for clones in 10 L bioreactor runs.

CHO-K1 Cell Clones	Cell Viability Post-Seeding (%)	Lowest Cell Viability Prior to Harvest (%)	Peak Ammonium Concentration (mM)	Day of Peak Production	Ammonium Concentration at Harvest (mM)	Day of Harvest
XA-a	97	88.8	7.22	9	5.21	14
XA-b	99	70.6	7.32	7	3.48	13
XA-c	94	69.1	5.17	5	2.49	11
XB-a	96	88.2	15.62	12	11.03	14
XB-b	99	74.9	9.10	7	8.8	13
XB-c	97	70.5	9.61	14	9.6	14

**Table 2:** Design power calculations for the Ambr^®^ 15 Cell Culture system for media evaluation.

	Delta (Signal)	Sigma (Noise)	Signal to Noise Ratio	Power for Media (%)	Power for Clone (%)
Titer (μg/mL)	2.8	0.9	3.1	99.9	99.0
Viability (%)	2	1	2	99.5	72.7

**Table 3:** Number of clones replicated in the three different media for evaluation of growth and productivity in Ambr 15 Cell Culture System.

Clone	XJEM	Dynamis AGT	EX-CELL Adv.
XA-a	2	2	2
XA-b	2	2	2
XA-c	2	2	2
XB-a	3	2	3
XB-b	2	2	2
XB-c	3	2	3

## Data Availability

The original contributions presented in this study are included in the article/Supplementary Materials. Further inquiries can be directed to the corresponding author.

## Supplementary Materials

The following supporting information can be downloaded at: https://www.mdpi.com/article/10.3390/toxins18050199/s1, Figure S1: WCX-HPLC of BoNT/A mAbs and BoNT/B mAbs; Figure S2: IEX-HPLC of individual mAbs; Figure S3. Overlay of HPLC chromatograms of each of the six individual BoNT/A and /B mAbs after dialysis in AEX Q FF equilibration buffer (25 mM citrate, 50 mM NaCl, pH 6.0). Blue: XA-b, black, XA-a; brown, XB-c, cyan, XB-a; green, XA-c; pink, XB-b. Detection was at 280nm; Figure S4. HPLC chromatogram of the mixture of the six mAbs (blue) compared to G03-52-01 drug product (black). The mAbs were dialyzed into the Capto Q column buffer A. Detection was at 280nm; Figure S5. Histogram comparing the production schedule and staff resources required for co-expression and purification vs. antibodies expressed singly. Based on the data shown in Table S9; Table S1. Antibody yield from Ambr co-expression and Protein A column; Table S2. Comparison of MabSelect PrismA vs. Fibro PrismA for Protein A capture; Table S3. Comparison of MabSelect PrismA vs. Fibro PrismA Protein A capture on product purity; Table S4. Comparison of Eshmuno CP-FT and Capto S CEX columns for recovery; Table S5. Comparison of anion exchange resins Capto Adhere MMC vs. Capto Q AEX; Table S6. Measured Fraction (1 mL per fraction) Concentrations by Nanodrop for CM Elution; Table S7. BioBLU^®^ 10c Fed-Batch Bioreactor Run Process Parameters; Table S8. Mobile phase gradient for ion exchange chromatography; Table S9. Comparison of production schedule and staff resources required for co-expression and purification vs. antibodies expressed singly.
