# Supplementary material for: Co-Expression and Co-Purification Enable Manufacturing of a Six-Monoclonal Antibody Botulinum Antitoxin Cocktail
Source: Toxins (Basel). 2026 Apr 23;18(5):199. doi: 10.3390/toxins18050199 (PMC13211578; doi:10.3390/toxins18050199)

Supplementary Materials

Table S1. Antibody yield from Ambr co-expression and Protein A column

Ambr mAb culture and media	Protein A HPLC Titer[1] (µg/mL)	Amount loaded onto Protein A column (mg)	Volume of Fraction Pool (mL)	Elution Amount (mg)	Yield (%)	Flowthrough (µg/mL)	Wash (µg/mL)
XA Co-expression-EX-CELL -1	774.13	3.5	5.6	2.70	77.6	28.65	0
XA Co-expression-EX-CELL -2	962.47	4.6	5.6	3.77	81.7	18.93	0
XA Co-expression-EX-CELL -3	995.03	4.5	4.4	3.40	75.8	0	0
XB Co-expression-EX-CELL -4	692.41	2.7	2.5	1.35	49.9	0	0
XB Co-expression-EX-CELL -5	627.17	2.3	3.1	1.47	63.5	0	0
XB Co-expression-EX-CELL -6	586.49	2.2	3.1	1.48	68.2	0	0
XB-a EX-CELL	1965.63	10.2	5.0	8.35	81.7	0	14.77
XB-b EX-CELL	1729.73	6.6	4.4	4.74	72.1	0	0
XB-c Dynamis-AGT	834.28	4.3	3.7	3.30	76.0	0	0
XB-c EX-CELL	169.39	1.0	1.9	0.53	53.9	0	0
XA-a EX-CELL	2033.62	11.6	6.2	8.22	70.9	0	0
XA-b EX-CELL	1521.72	8.8	5.6	7.70	87.2	0	15.8
XA-c EX-CELL	1065.63	5.3	4.4	4.59	86.2	0	0

[1] HPLC with detection at A280 nm was used to calculate the yield.

Table S2. Comparison of MabSelect PrismA vs. Fibro PrismA for Protein A capture

Clone	Titer (g/L)	MabSelect PrismA (5mL column)			Fibro PrismA (0.4 mL Fibro unit)		
		Relative Clearance (g/L)	Recovery (%)	Productivity (g/L/h)	Relative Clearance (g/L)	Recovery (%)	Productivity (g/L/h)
XA-a	0.70	49	82	10	40	99	278
XA-b	1.70	52	87	18	41	103	288
XA-c	0.74	55	91	11	36	89	243
XB-a	1.26	54	90	16	36	90	252
XB-b	1.86	58	97	18	37	93	296
XB-c	1.07	50	83	12	32	87	224
Average	1.22	53	88.33	14.17	37	93.5	263.5

Table S3. Comparison of MabSelect PrismA vs Fibro PrismA Protein A capture on product purity

MabSelect PrismA	Fibro PrismA

Clone	Host cell protein (ng/mL)	Relative Clearance	Total Nucleic Acid (ng/mL)	Relative Nucleic Acid Clearance	Host cell protein (ng/mL)	Relative Host cell protein Clearance	Total Nucleic Acid (ng/mL)	Relative Nucleic Acid Clearance
XA-a	N.M.	N.M.	206	151	N.M.	N.M.	105	297
XA-b	11,069	8	279	100	7,056	12	176	158
XA-c	12,446	30	293	57	5,265	70	310	54
XB-A	21,580	13	229	26	14,258	20	101	59
XB-b	59,259	5	N.M.	N.M.	7,422	21	176	N.M.
XB-c	17,128	29	428	51	5,902	84	283	78

N.M. : not measured

Table S4. Comparison of Eshmuno CP-FT and Capto S CEX columns for recovery

	Eshmuno CP-FT				Capto S			
Clone	Load (g/L)	Recovery (%)	Productivity (g/L/h)	Residual total Nucleic Acid (ng/mL)	Load (g/L)	Recovery (%)	Productivity (g/L/h)	Residual total Nucleic Acid (ng/mL)
XA-a	790	78	154	7	N.T.	N.T.	N.T.	N.T.
XA-b	1,000	87	215	122	657	86	141	139
XA-c	N.T.	N.T.	N.T.	N.T.	882	81	238	121
XB-A	690	87	225	65	644	77	180	58
XB-b	1,000	95	266	130	539	78	158	46
XB-c	1,000	85	197	34	N.T.	N.T.	N.T.	N.T.
Average ± S.D.	896 ± 147	86 ± 6	211 ± 41	72 ± 54	681 ± 144	81± 4	179 ± 42	91 ± 46

N.T.: not tested

Table S5. Comparison of anion exchange resins Capto Adhere MMC vs. Capto Q AEX

	Capto Adhere				Capto Q			
Clone	Load (g/L)	Recovery (%)	Productivity (g/L/h)	Total Nucleic Acid (ng/mL)	Load (g/L)	Recovery (%)	Productivity (g/L/h)	Total Nucleic Acid (ng/mL)
XA-a	276	79	79	80	293	102	99	80
XA-b	688	81	147	110	630	100	157	130
XA-c	315	94	111	70	296	97	96	70
XB-A	460	98	159	170	474	96	143	170
XB-b	590	97	191	210	622	97	190	240
XB-c	382	95	121	30	422	98	131	40
Average ± SD	452 ± 161	91 ± 8	135 ± 39	112 ± 67	456 ± 149	98.3 ± 2.3	136 ± 36	122 ± 74

Table S6. Measured Fraction (1 mL per fraction) Concentrations by Nanodrop for CM Elution

Sample	Concentration (mg/mL)
Load	0.511
CM Flow through	0

CM wash out	0
CM12	0.014
CM13	0.276
CM14	1.000
CM15	2.219
CM16	2.834
CM17	2.044
CM18	1.031
CM19	0.404
CM20	0.150
CM21	0.062
CM22	0.021
CM23	0.009

Table S7. BioBLU® 10c Fed-Batch Bioreactor Run Process Parameters

**Parameter**	**Target Value**
Agitation	120 rpm
Temperature	36.5°C shifted to 32°C at viable cell density plateau
Dissolved Oxygen (DO)	40%
Air Flow (3-Gas Mix)	0.06 – 0.04 liters per minute at standard temperature and pressure
pH	7.1 ± 0.2
Acid Control	10% Mix CO_2
Base Control	Pump 2: 5 mL/min with 1M Sodium bicarbonate
Glucose Feed concentration	5.5 – 8.0 g/L
Glutamine Feed concentration	4 mM
Basal Medium	6 L initial working volume<br>(5 L EX-CELL® Media + 1 L Cell Inoculum)
Nutrient Feed	0.45 L on Days 3, 5, 7, 9, 11, 13
Seeding Density of cell clones	0.4 x 10^6 Vc/mL
Day of Harvest	Day 11 – 14 or when viability went below 70%

Table S8. Mobile phase gradient for ion exchange chromatography

**Time (minutes)**	**Mobile Phase A**	**Mobile Phase B**
0	100	0
2	98	2
17	93	7
25	93	7
40	88	12
70	80	20
73	70	30
73.1	60	40
78	60	40
78.1	100	0

88	100	0

Table S9. Comparison of production schedule and staff resources required for co-expression and purification vs. antibodies expressed singly.

WEEK	1	2	3	4	5	6	7	8	9	10	11	12	13	14	15	16
3 separate mAb expression/purification																
mAb 1	scale up			production		purification		testing		document-ation						
FTEs required	1	1	2	4	4	4	4	4	4	2						
mAb 2				scale up			production		purification		testing		documentation			
FTEs required				1	1	2	4	4	4	4	4	4	2			
mAb 3							scale up			production		purification		testing		documentation
FTEs required							1	1	2	4	4	4	4	4	4	2
**Total FTE-weeks for separate production**	1	1	2	5	5	6	9	9	10	10	8	8	6	4	4	2
3 mAbs combined expression/purification																
	scale up			production		purification		testing		documentation						
**Total FTE-weeks for combined approach**	3	3	6	4	4	4	4	4	4	2						

Timeline assumes that only one production suite is available, requiring a staggered start of production, and that cell culture expansion from 5 to 10 L can be performed in a single suite.

Abbreviation: FTE, full-time equivalent employee

Figure S1. Weak cation-exchange HPLC of three-antibody BoNT/A mAbs and BoNT/B mAbs

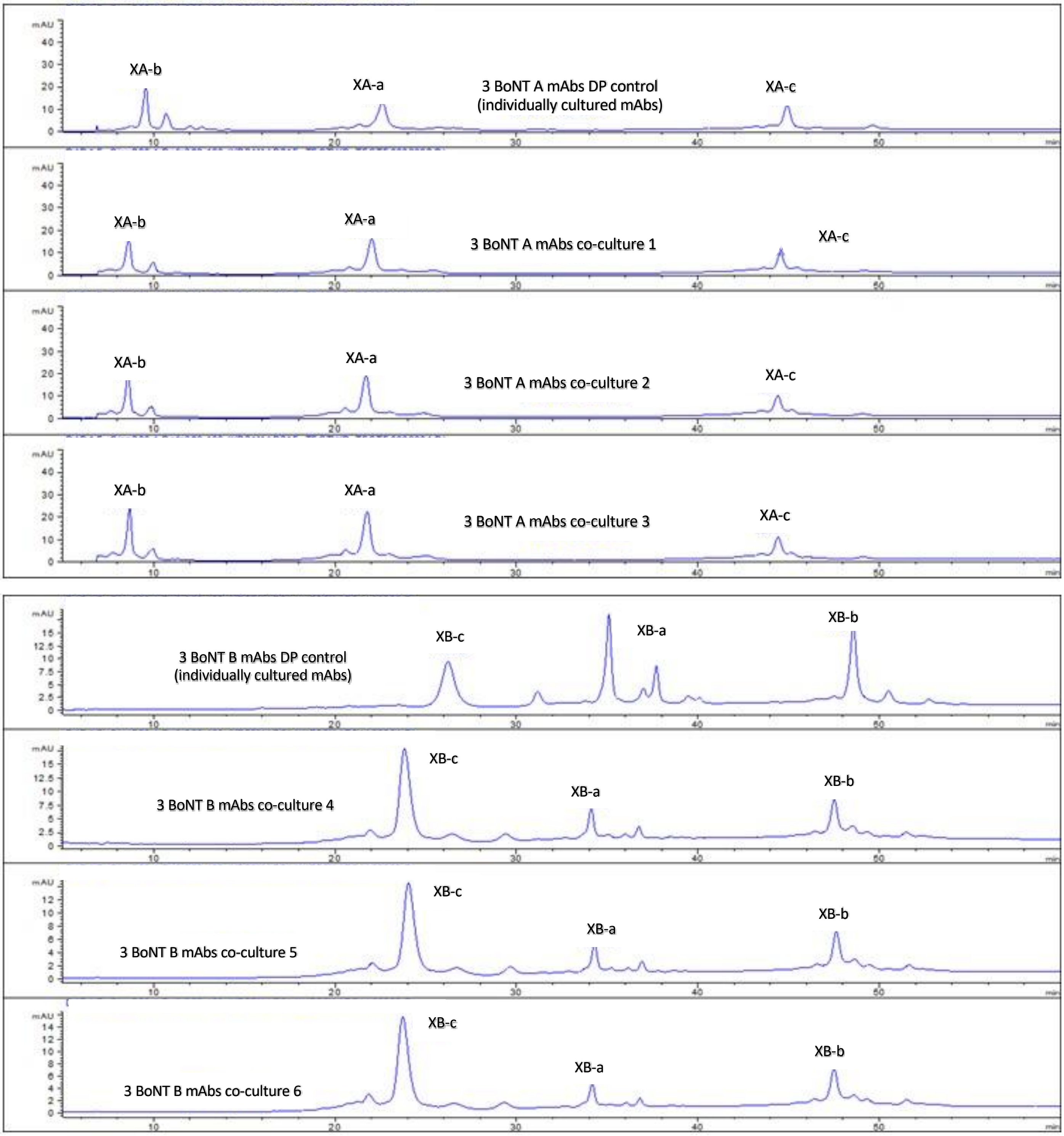

Figure S2.
IEX-HPLC of individual mAbs

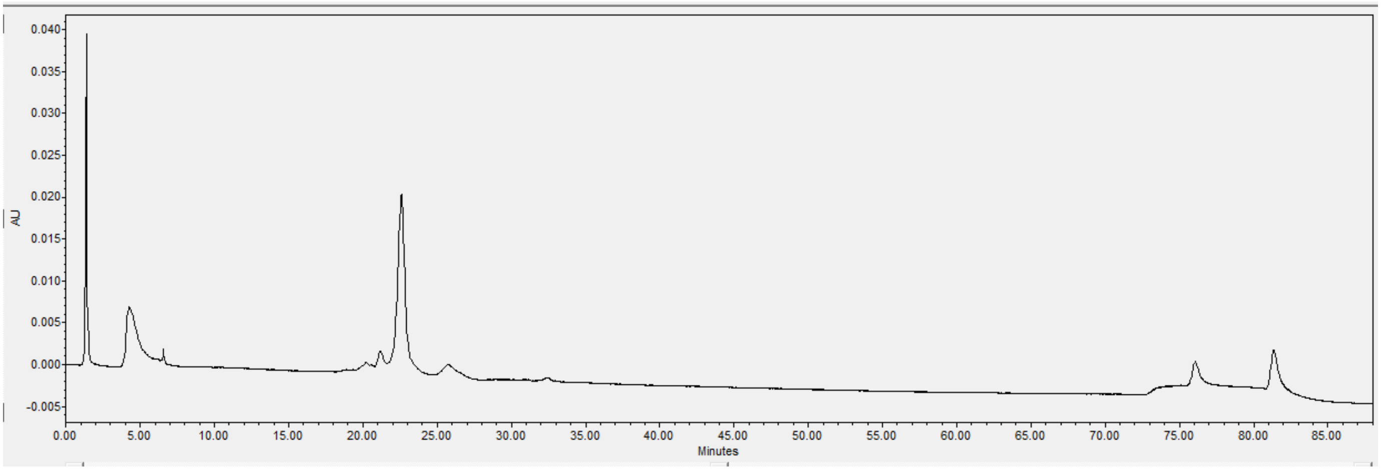
AU
Minutes
0.040
0.035
0.030
0.025
0.020
0.015
0.010
0.005
0.000
-0.005
0.00 5.00 10.00 15.00 20.00 25.00 30.00 35.00 40.00 45.00 50.00 55.00 60.00 65.00 70.00 75.00 80.00 85.00

XA-a

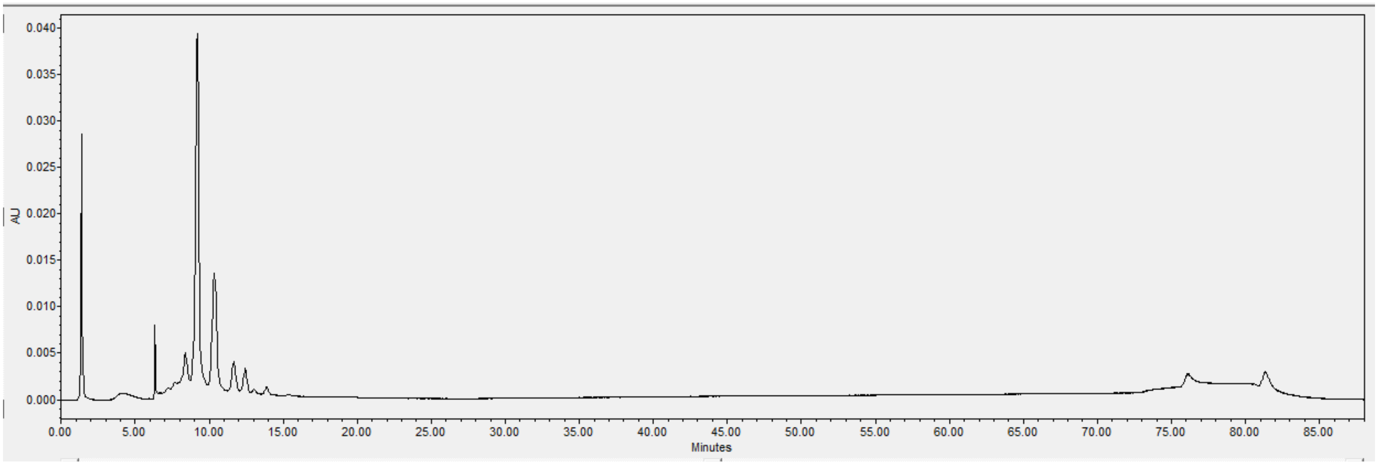
AU
Minutes
0.040
0.035
0.030
0.025
0.020
0.015
0.010
0.005
0.000
0.00 5.00 10.00 15.00 20.00 25.00 30.00 35.00 40.00 45.00 50.00 55.00 60.00 65.00 70.00 75.00 80.00 85.00

XA-b

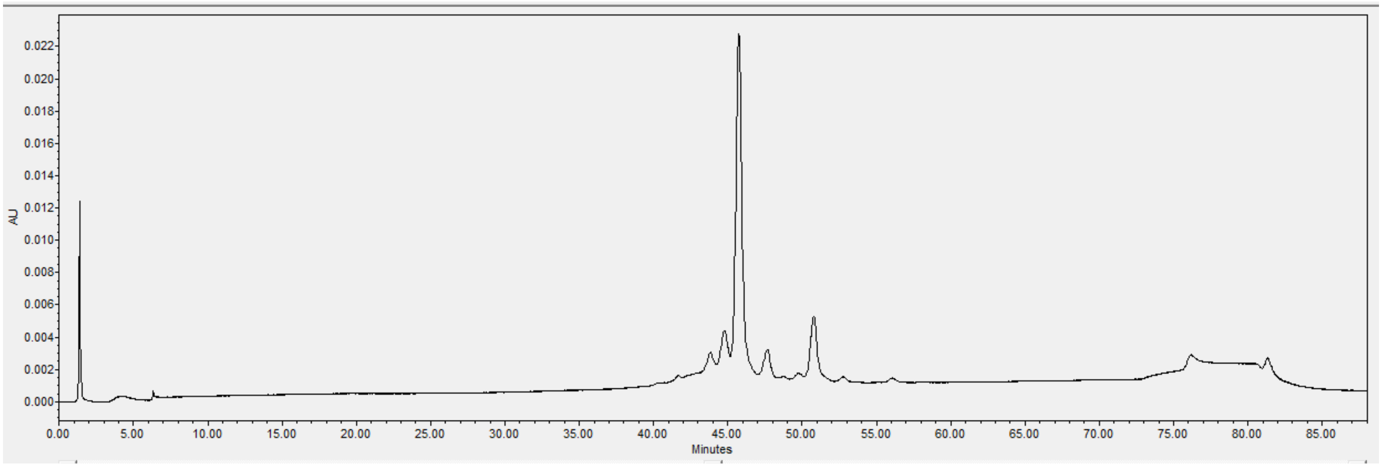
AU
Minutes
0.022
0.020
0.018
0.016
0.014
0.012
0.010
0.008
0.006
0.004
0.002
0.000
0.00 5.00 10.00 15.00 20.00 25.00 30.00 35.00 40.00 45.00 50.00 55.00 60.00 65.00 70.00 75.00 80.00 85.00

XA-c

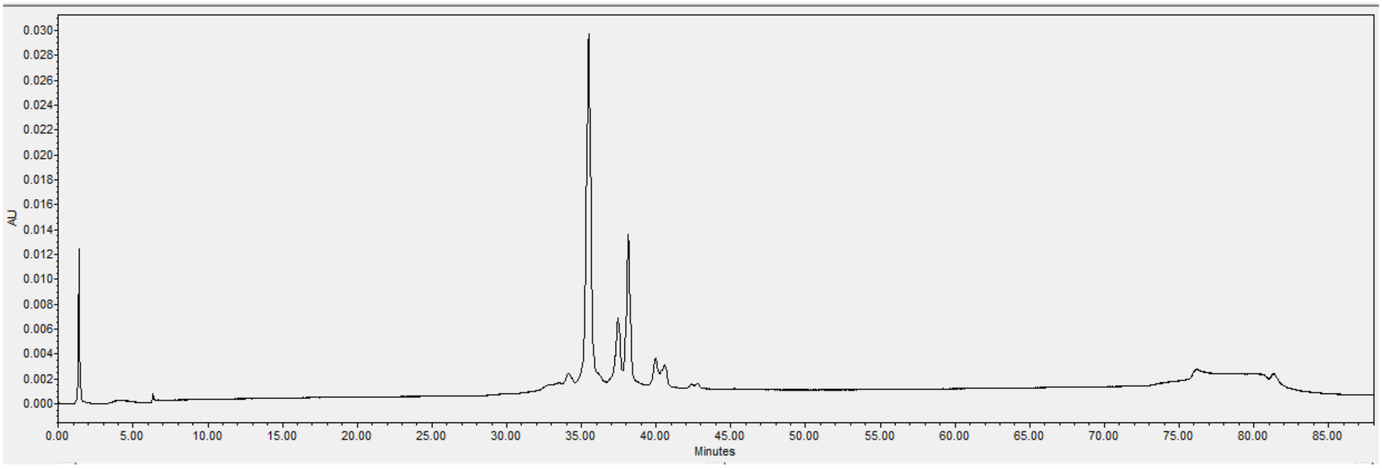
AU
0.000 0.002 0.004 0.006 0.008 0.010 0.012 0.014 0.016 0.018 0.020 0.022 0.024 0.026 0.028 0.030
0.00 5.00 10.00 15.00 20.00 25.00 30.00 35.00 40.00 45.00 50.00 55.00 60.00 65.00 70.00 75.00 80.00 85.00
Minutes
XB-a

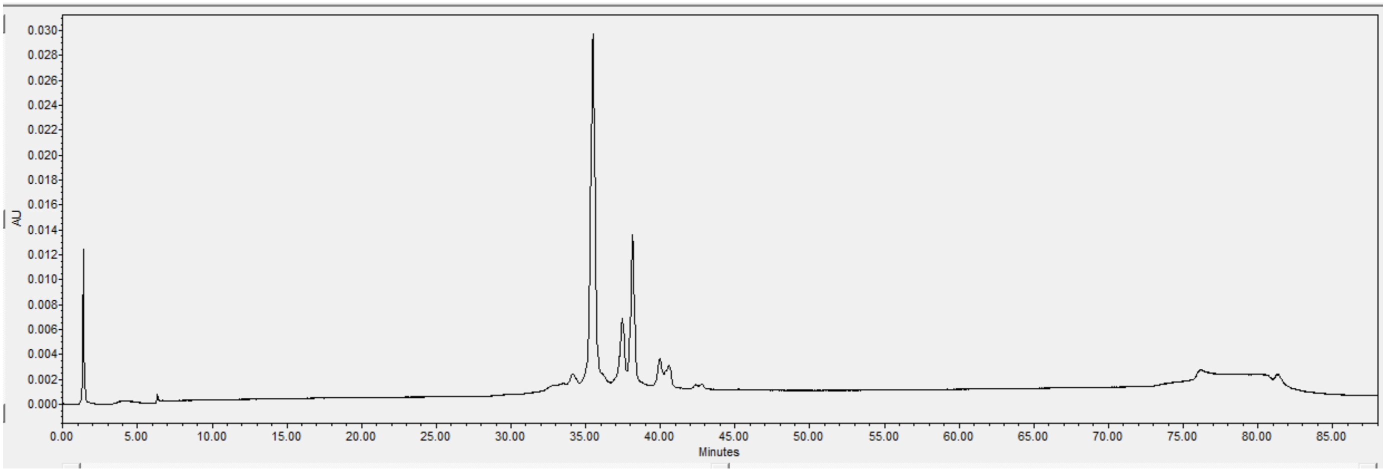
AU
0.000 0.002 0.004 0.006 0.008 0.010 0.012 0.014 0.016 0.018 0.020 0.022 0.024 0.026 0.028 0.030
0.00 5.00 10.00 15.00 20.00 25.00 30.00 35.00 40.00 45.00 50.00 55.00 60.00 65.00 70.00 75.00 80.00 85.00
Minutes
XB-b

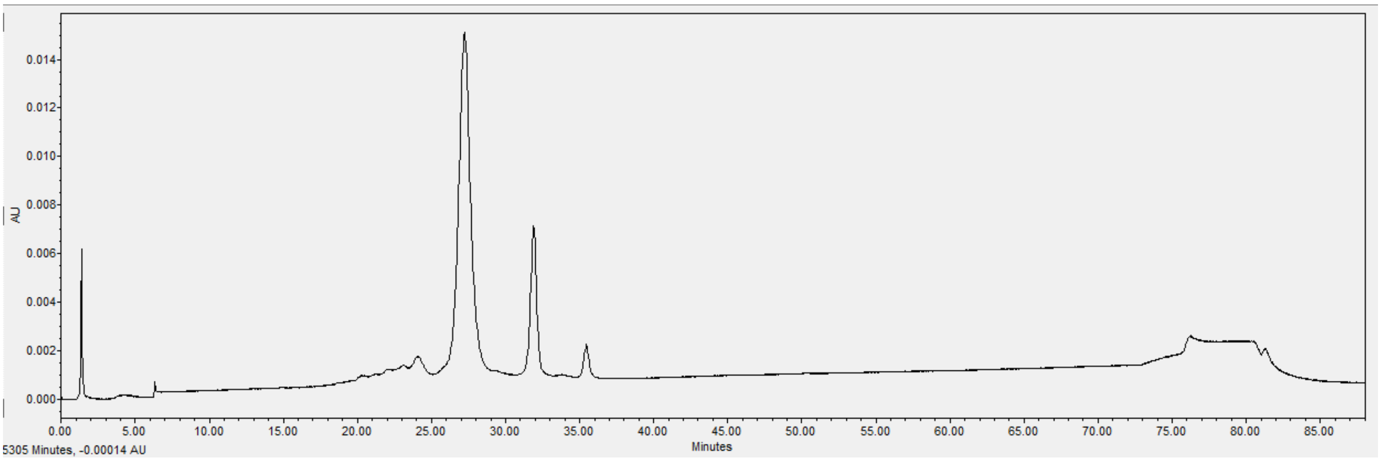
AU
0.000 0.002 0.004 0.006 0.008 0.010 0.012 0.014
0.00 5.00 10.00 15.00 20.00 25.00 30.00 35.00 40.00 45.00 50.00 55.00 60.00 65.00 70.00 75.00 80.00 85.00
Minutes
5305 Minutes, -0.00014 AU
XB-c

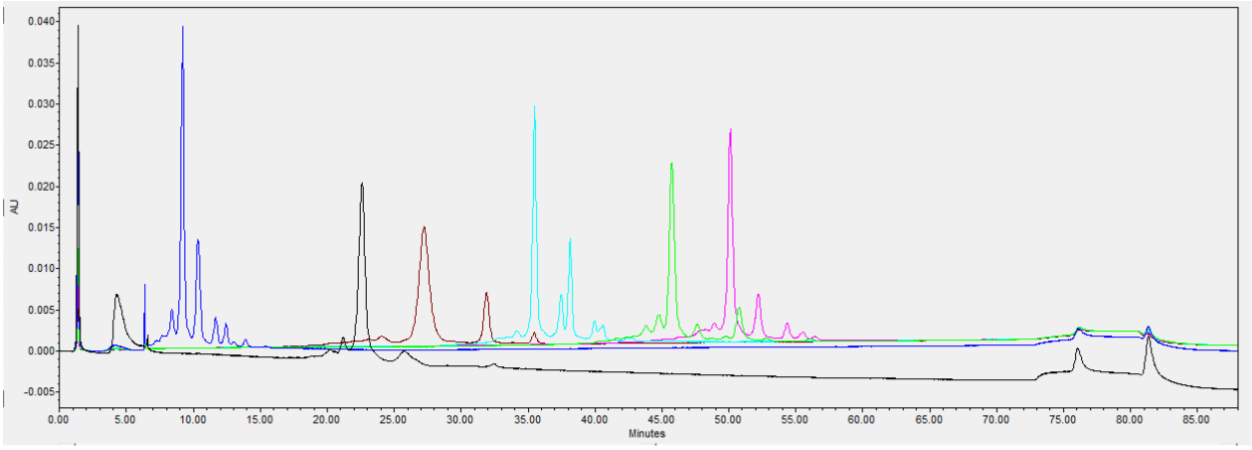

Figure S3. Overlay of HPLC chromatograms of each of the six individual BoNT/A and /B mAbs after dialysis in AEX Q FF equilibration buffer (25 mM citrate, 50 mM NaCl, pH 6.0). Blue: XA-b, black, XA-a; brown, XB-c, cyan, XB-a; green, XA-c; pink, XB-b. Detection was at 280nm.

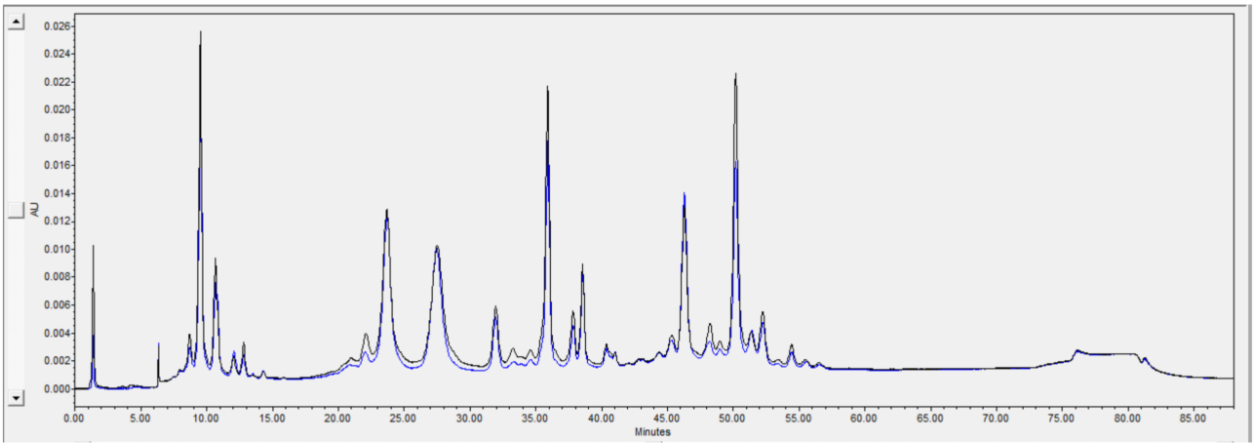

Figure S4. HPLC chromatogram of the mixture of the six mAbs (blue) compared to G03-52-01 drug product (black). The mAbs were dialyzed into the Capto Q column buffer A. Detection was at 280nm.

Figure S5. Histogram comparing the production schedule and staff resources required for co-expression and purification vs. antibodies expressed singly. Based on the data shown in Table S9.

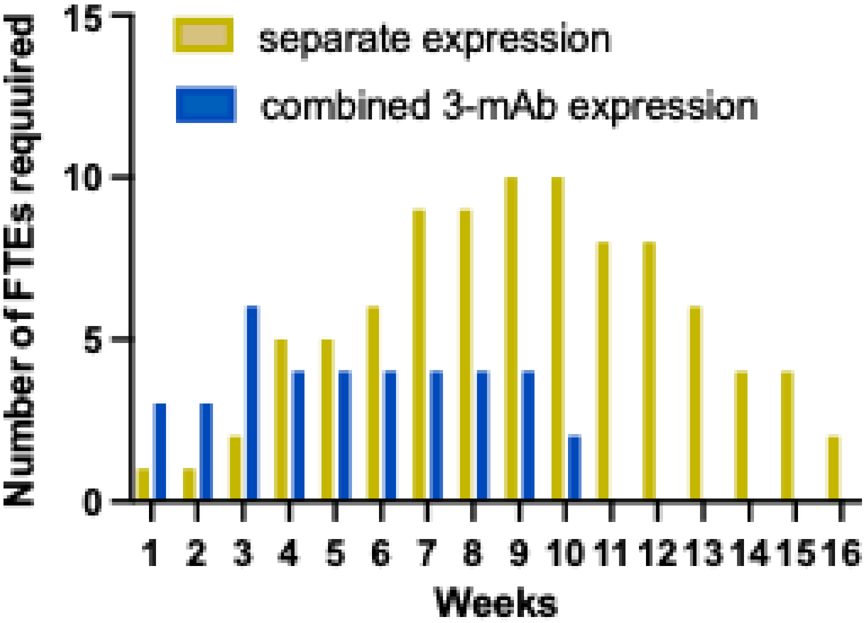

Supplement: Supplementary file 1 [file toxins-18-00199-s001.zip › toxins-4113757-supplementary.pdf]
